# Diverse Molecular Mechanisms Underlying Microbe-Inducing Male Killing in the Moth Homona magnanima

**DOI:** 10.1128/aem.02095-22

**Published:** 2023-04-26

**Authors:** Hiroshi Arai, Takumi Takamatsu, Shiou-Ruei Lin, Tetsuya Mizutani, Tsutomu Omatsu, Yukie Katayama, Madoka Nakai, Yasuhisa Kunimi, Maki N. Inoue

**Affiliations:** a United Graduate School of Agricultural Science, Tokyo University of Agriculture and Technology, Fuchu, Tokyo, Japan; b National Agriculture and Food Research Organization (NARO), Tsukuba, Ibaraki, Japan; c Tea Research and Extension Station, Council of Agriculture, Yangmei, Taoyuan, Taiwan; d Center for Prevention of Global Infectious Diseases of Animals, Faculty of Agriculture, Tokyo University of Agriculture and Technology, Fuchu, Tokyo, Japan; Norwegian University of Life Sciences

**Keywords:** *Spiroplasma*, *Wolbachia*, endosymbionts, male killing, Partitiviridae, symbiosis

## Abstract

Male killing (MK) is a type of reproductive manipulation induced by microbes, where sons of infected mothers are killed during development. MK is a strategy that enhances the fitness of the microbes, and the underlying mechanisms and the process of their evolution have attracted substantial attention. Homona magnanima, a moth, harbors two embryonic MK bacteria, namely, *Wolbachia* (*Alphaproteobacteria*) and *Spiroplasma* (*Mollicutes*), and a larval MK virus, Osugoroshi virus (OGV; Partitiviridae). However, whether the three distantly related male killers employ similar or different mechanisms to accomplish MK remains unknown. Here, we clarified the differential effects of the three male killers on the sex-determination cascades and development of *H. magnanima* males. Reverse transcription-PCR demonstrated that *Wolbachia* and *Spiroplasma*, but not OGVs, disrupted the sex-determination cascade of males by inducing female-type splice variants of *doublesex* (*dsx*), a downstream regulator of the sex-determining gene cascade. We also found that MK microbes altered host transcriptomes in different manners; *Wolbachia* impaired the host dosage compensation system, whereas *Spiroplasma* and OGVs did not. Moreover, *Wolbachia* and *Spiroplasma*, but not OGVs, triggered abnormal apoptosis in male embryos. These findings suggest that distantly related microbes employ distinct machineries to kill males of the identical host species, which would be the outcome of the convergent evolution.

**IMPORTANCE** Many microbes induce male killing (MK) in various insect species. However, it is not well understood whether microbes adopt similar or different MK mechanisms. This gap in our knowledge is partly because different insect models have been examined for each MK microbe. Here, we compared three taxonomically distinct male killers (i.e., *Wolbachia*, *Spiroplasma*, and a partiti-like virus) that infect the same host. We provided evidence that microbes can cause MK through distinct mechanisms that differ in the expression of genes involved in sex determination, dosage compensation, and apoptosis. These results imply independent evolutionary scenarios for the acquisition of their MK ability.

## INTRODUCTION

Male killing (MK), the phenomenon of male death during development, is caused by microbes, such as intracellular bacteria, microsporidia, and viruses, in various insects (1–3). MK is classified by its timing of action; those typically occurring during embryogenesis, or early larval stages, are referred to as early MK, while those typically occurring during matured larval stages are designated late MK (1–4). As male killers directly lead to female-biased sex ratios in insects, MK is a selfish strategy that promotes the inheritance and propagation of maternally inherited microbes in nature (4). Moreover, male killers can have an overall advantage if the female hosts benefit from the death of their male siblings (4–6). The primary advantage associated with early MK is the reallocation of resources to female insects (transmitting sex) that were to be consumed by males. For example, the ladybug beetle Adalia bipunctata harbors phylogenetically distinct early MK bacterial species (7–9). MK bacteria, such as *Wolbachia* and *Spiroplasma* enhance the fitness of infected females by exploiting the behavior of ladybugs and encouraging them to feed on their dead brothers (6–9). In contrast, the advantage of late MK induced by microsporidia (10) or viruses (11, 12) is the maximization of male-killer density in the host allowing for horizontal transmission.

**FIG 1:**
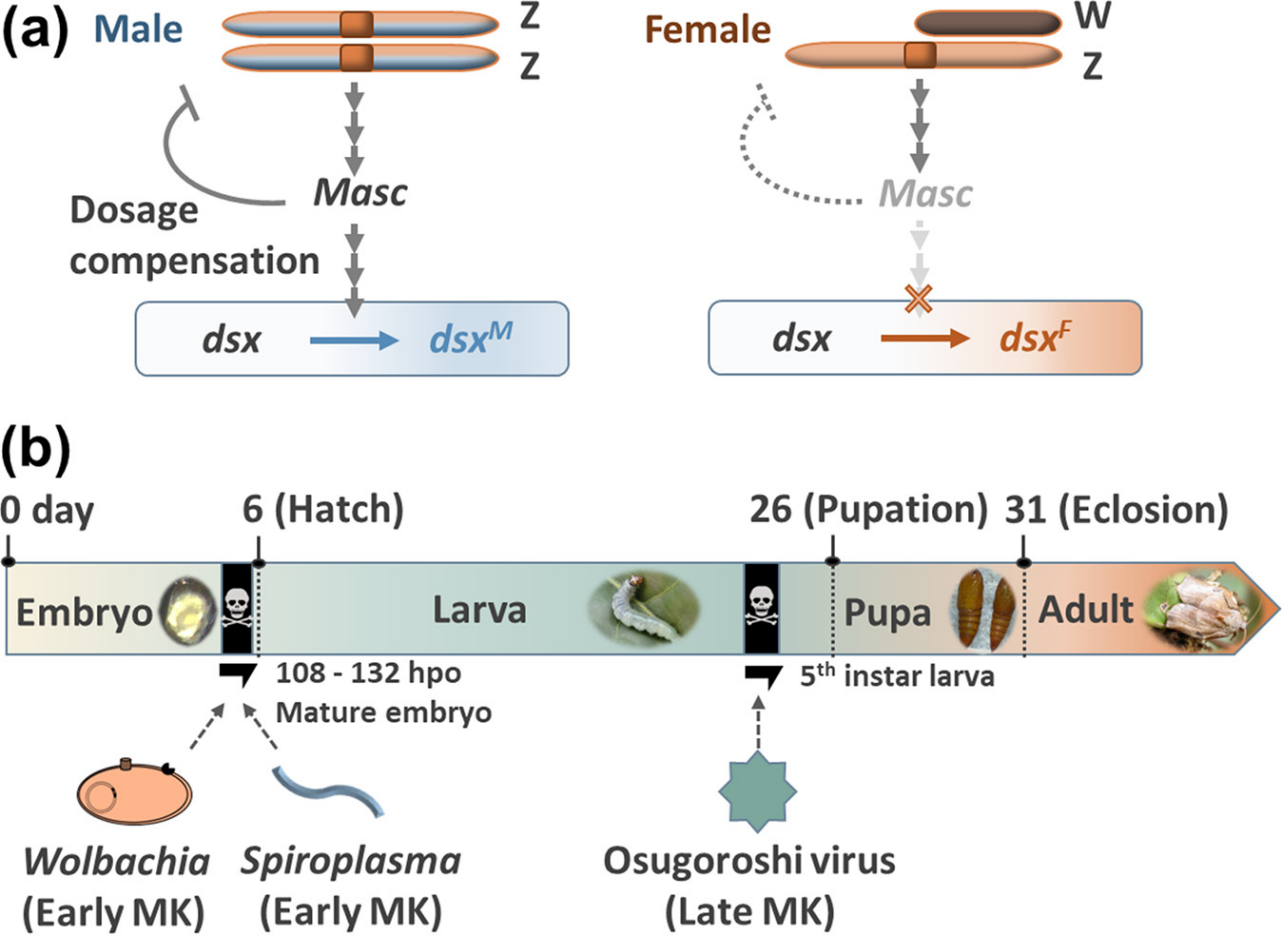
Sex-determination cascade and lethal stages of male killers in *H. magnanima*. (a) Model of the sex-determination cascade in lepidopteran insects based on a previous report by Kiuchi et al. (30). *Masc* facilitates the expression of the male-type *dsx* splicing. Z, Z chromosome; W, W chromosome. (b) Lethal stages of male killers in *H. magnanima*. Both *Wolbachia* and *Spiroplasma* kill males during the mature embryonic stage. Osugoroshi viruses (OGVs) induce late MK during the larval stage. hpo, hour postoviposition.

A key question is how phylogenetically distinct microbes evolved similar MK phenotypes. Recent studies have indicated that MK could involve diverse mechanisms owing to complex interactions between bacteria and host species (13–23). In theory, any molecular machineries involved in sex determination and differentiation could be targeted by MK microbes (2, 5, 13–16). Many insects employ the sex chromosome dosage compensation system that balances sex-linked gene expression between males and females. Different organisms deploy distinct strategies to achieve dosage compensation. In insects that harbor XX/XY sex chromosome systems (e.g., *Drosophila* flies), transcriptional upregulation of the single X chromosome of XY males equalizes its output to that from two X copies of XX females (24–26). In Lepidoptera harboring the ZW/ZZ sex chromosomes, dosage compensation involves the downregulation of Z-chromosome genes in ZZ males so that they can achieve balanced expression with the single Z in females (27–29). In lepidopteran insects, such as Bombyx mori and *Ostrinia* moths, the protein Masculinizer (Masc) regulates the dosage compensation as well as male sex determination (Fig. 1a) (30, 31). In *Ostrinia* moths, the MK *Wolbachia* strains *w*Fur and *w*Sca degrade Masc, which disrupts dosage compensation and alters the splicing pattern of the core organizing gene for sexual differentiation *doublesex* (*dsx*) (14–16). In contrast, the *w*Bif strain of *Wolbachia* in Drosophila bifasciata and Spiroplasma poulsonii (Mollicutes) in D. melanogaster induce male-specific abnormal apoptosis by damaging the dosage-compensated chromosome (X chromosome) of the male without affecting the sex-determination cascade (17–19). Although MK *Wolbachia* and *Spiroplasma* cause similar DNA damage in *Drosophila* males, they use different genes, with *S. poulsonii* using the MK toxin called Spaid and *Wolbachia* using an MK candidate gene called *wmk* (18, 22, 23). However, it remains unclear to what extent male killers share their MK mechanisms.

Here, we report that distantly related three male killers exert MK via different mechanisms in the tea tortrix moth Homona magnanima (Tortricidae, Lepidoptera). Previously, we demonstrated that *Wolbachia* strain *w*Hm-t (23, 32) and Spiroplasma ixodetis *s*Hm (33, 34) cause early MK (embryonic MK); however, the partiti-like Osugoroshi virus (OGV) causes late MK (larval MK) in *H. magnanima* (11, 12, 35, 36). By comparing the effects of microbes at critical time points where MK occurs (Fig. 1b), we confirmed that the previously mentioned MK microbes affect the dosage compensation system, sex-determination cascades, and host development via different mechanisms. In addition, we discuss the origin and evolution of MK mechanisms induced by various microbes.

## RESULTS AND DISCUSSION

### MK *Wolbachia* and *Spiroplasma*, but not OGVs, altered the splicing patterns of *dsx* in males.

**FIG 2:**
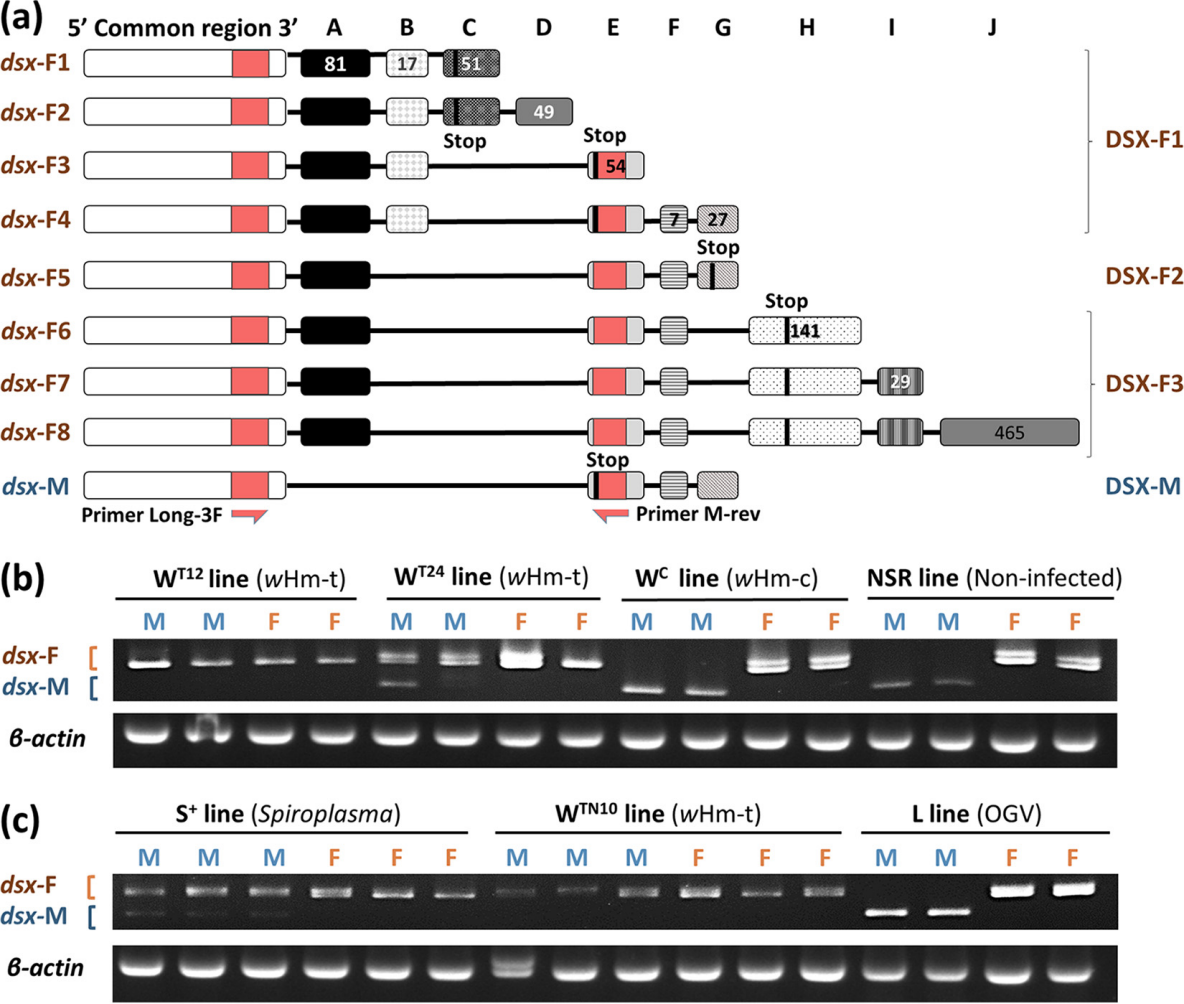
Effects of male killers on *doublesex* (*dsx*) mRNA splicing in *H. magnanima*. (a) Nine *dsx* splicing variants in *H. magnanima*. Females had eight *dsx* variants (*dsx*-F1 to *dsx*-F8) encoding three DSX proteins (DSX-F1 to 3), and males showed a single variant (DSX-M). Rectangular boxes represent exons (A to J). The numbers inside the boxes indicate the numbers of base pairs. Stop codons in each variant are indicated with black or white bars. Primer binding regions are shown with a solid red color. (b) *dsx*-splicing patterns in hosts harboring *w*Hm-t (W^T12^ and W^T24^) or *w*Hm-c (W^C^) and NSR lines. (c) *dsx*-splicing patterns in hosts harboring *Spiroplasma* (S^+^), *w*Hm-t (W^TN10^), or OGVs (L). *dsx-M*, male-type *dsx* (377 bp); *dsx-F*, female-type *dsx* (457 bp and 471 bp). β-actin was amplified as a control gene.

To evaluate the effects of the three male killers on sex determination in *H. magnanima*, we first determined the *dsx* transcript sequences. Insects frequently exhibit sex-specific *dsx* splicing variants, referred to as *dsx*-M (male type) and *dsx*-F (female type) (15, 37–39). According to rapid-amplification of cDNA ends (RACE) assays using an *H. magnanima* normal-sex ratio line (NSR), males showed a variant *dsx*-M encoding the protein DSX-M, whereas females had eight splicing variants (*dsx*-F1 to 8) encoding three proteins (DSX-F1 to 3) (Fig. 2a). We then designed a primer pair to distinguish the *H. magnanima dsx* splicing variants *dsx*-M and *dsx*-F (type 3 to 8, Fig. 2a) using a diagnostic PCR (forward primer in coding sequence and reverse primer in noncoding region Exon E). Male embryos (at 108 h postoviposition [hpo] when early MK occurs [32, 34] [Fig. 1]) infected with either early MK *w*Hm-t (W^T12^, W^T24^, and W^TN10^) or *Spiroplasma s*Hm (S+) exhibited *dsx*-F, whereas male embryos of the NSR line, L line harboring OGVs, and the W^c^ line harboring non-MK *Wolbachia w*Hm-c (40) showed only *dsx*-M (Fig. 2b and c). In addition, S+ and W^T24^ males exhibited both *dsx*-M and *dsx*-F (Fig. 2b and c). Bacterial density is an important factor determining the expression of MK induced by *Wolbachia* and *Spiroplasma* (32, 34, 41). Previously, we have shown that W^T24^ lines exhibit higher hatchability and lower *w*Hm-t amounts than the W^T12^ lines (32). The appearance of both *dsx*-M and *dsx*-F in embryos suggests that bacterial densities were not enough to completely alter the *dsx* splicing. Moreover, OGVs did not alter *dsx* splicing in embryos as well as in moribund male larvae (5th instar) showing symptoms of OGV infection (i.e., carcinoma-like tissue) (see Fig. S1 in the supplemental material).

Previous studies have shown that MK *Wolbachia* strains *w*Fur and *w*Sca alter *dsx* splicing patterns in male embryos of *Ostrinia* moths (14–16). In the present study, we highlighted that MK *Wolbachia w*Hm-t and *Spiroplasma s*Hm, but not OGVs, induced female-type *dsx* splicing in males of *H. magnanima*. We also confirmed that non-MK strain *w*Hm-c, which is closely related to the MK strain *w*Hm-t (32), did not alter *dsx* splicing. We recently discovered an MK-associated prophage region that was present in *w*Hm-t but absent in *w*Hm-c by comparing their genomes (23). The prophage region encodes a homolog of the protein Oscar that recapitulates *w*Fur-induced MK and alters *dsx* splicing in *Ostrinia* moths (42). The *Wolbachia* strain *w*Hm-t carries phage genes that may disrupt the sex-determination cascades of *H. magnanima* males. We speculate that MK *Wolbachia* generally interferes with the male’s sex-determination cascades in Lepidoptera. In contrast, to our knowledge, it has not been reported that MK *Spiroplasma* interferes with the host’s sex-determination cascades, including *Drosophila* (17–20). The presence or absence of the altered *dsx* splicing in *H. magnanima* and *Drosophila* could be due to the difference in host genetic backgrounds (i.e., Diptera and Lepidoptera) and/or the difference in bacterial species (i.e., *S. poulsonii* and *S. ixodetis*). In addition, no homologous genes were found in the *S. ixodetis s*Hm genome and the MK-associated prophage region of *w*Hm-t (23, 34), suggesting that *s*Hm and *w*Hm-t alter *dsx* splicing in males via different mechanisms.

### Early MK *Wolbachia* impaired dosage compensation in males.

The dosage compensation system adjusts expression levels of sex chromosome genes between males and females (24–29). In the family Tortricidae (Lepidoptera), males have two Z chromosomes (ZZ) and show equivalent expression levels of Z-linked genes as those in females (ZW) (29, 43–47). In contrast to *B. mori* and *Ostrinia* moths, Tortricidae moths, including *Homona*, generally contain a large Z chromosome consisting of homologs of *B. mori* chromosome 1 (Z chromosome) and chromosome 15 (autosome) (29, 43–47). Considering that *Wolbachia* infection results in male-specific embryonic lethality due to a failure of dosage compensation in *Ostrinia* moths (14, 42), we hypothesized that microbes in *H. magnanima* accomplish MK by causing a failure of dosage compensation via Z-linked gene overexpression in males. We evaluated the effects of male killers on dosage compensation in *H. magnanima* embryos (108 hpo) (Fig. 1) by RNA-sequencing (RNA-seq). Among the *H. magnanima de novo* assembled data (293,111 contigs; mean length, 868.3 bp; total length, 254,508,797 bp), 54,071 contigs were annotated to the *B. mori* genes. The fold change in the expression (transcripts per million [TPM]) of each annotated contig between males and females was calculated and plotted on the corresponding *B. mori* chromosome. As reported previously in *Wolbachia*-infected *Ostrinia* (14), putative Z-linked genes (i.e., *H. magnanima* genes corresponding to genes on *B. mori* chromosome 1 and 15) were expressed at higher levels in *w*Hm-t-infected *H. magnanima* males than those in females. In contrast, expression levels of putative Z-linked genes were equivalent between males and females in the NSR, *s*Hm-infected, and OGVs-infected lines. These results suggest that only *w*Hm-t affects the dosage compensation system of *H. magnanima* during the embryogenesis stage.

**FIG 3:**
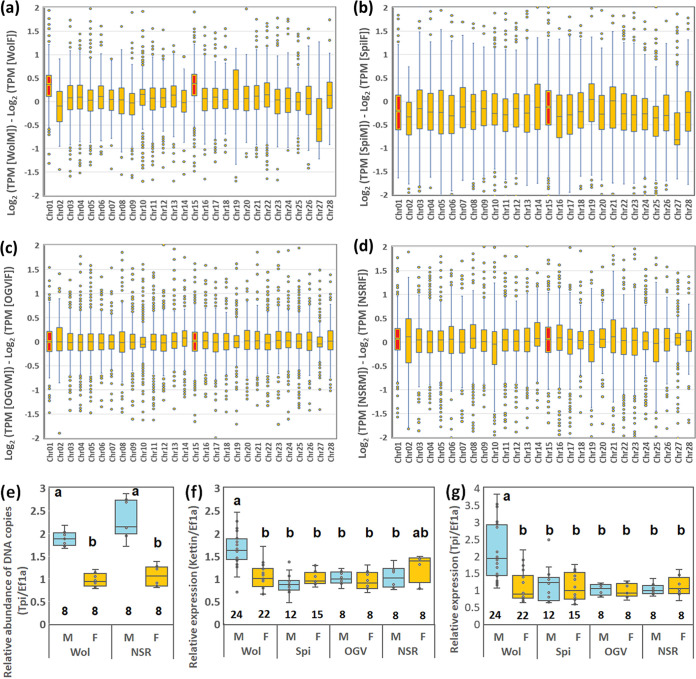
Male killers affected *H. magnanima* dosage compensation differently. (a to d) Normalized expression levels (TPM) and chromosomal distributions of transcripts in *H. magnanima* embryos. RNA-seq data (108 hpo) were used to make the following comparisons: W^T12^ males versus W^T12^ females (a), S^+^ males versus S^+^ females (b), L males versus L females (c), and NSR males versus NSR females (d). The chromosome number for each *H. magnanima* transcript-derived contig was assigned based on *B*. *mori* gene models. The boxes in the box-and-whisker diagrams represent the median and 25 to 75 percentile ranges of the expression ratios. (e to g) Quantification of Z-linked genes in *H. magnanima* embryos (108 hpo). The *Tpi* gene dose (e) was quantified to assess whether males have two Z chromosomes. *Kettin* (f) and *Tpi* (g) expression levels were normalized to that of the autosomal *Ef1α* gene. Error bars represent the standard error. The *y* axis indicates relative abundances adjusted to those of NSR females. Different letters indicate significant differences determined by the Steel-Dwass test (*P < *0.05). The numbers inside the bars indicate replicates. Wol, Wolbachia wHm-t, WT12 line; Spi, Spiroplasma sHm, S^+^ line; OGV, L line; NSR, NSR line.

To confirm whether MK microbes affect the male dosage compensation system differently, we further quantified the expression of conserved Z-linked genes triosephosphate isomerase (*HmTpi*) and kettin (*HmKettin*), which are homologs of the genes on *B. mori* chromosome 1, using quantitative PCR (qPCR) assays. The *HmTpi* gene dose was 2-fold higher in males than that in females, regardless of *Wolbachia* infection, confirming that males had homogametic sex chromosomes (ZZ), whereas females had heterogametic sex chromosomes (ZW) (Fig. 3e). Moreover, the Z-linked genes *HmTpi* and *HmKettin* were expressed at 2-fold higher levels in male embryos (108 hpo) harboring *w*Hm-t than those in females (Steel-Dwass test, *P < *0.005). In contrast, *Spiroplasma* and OGV infection did not affect gene expression levels between the sexes (*Spiroplasma*, *P* = 0.65 for *HmTpi* and *P *= 1.00 for *HmKettin*; OGV, *P* = 0.99 for *HmTpi* and *P* = 0.99 for *HmKettin*) (Fig. 3f and g). These results confirmed that only *w*Hm-t disrupts the male dosage compensation system during the embryogenesis stage. We also assessed whether OGVs affect dosage compensation during the larval stages. Although the samples were limited in number, the OGV-infected moribund male larvae (5th instar) did not show apparent fold changes in the Z-linked genes compared with females (Fig. S1) (Steel-Dwass test, *P* = 0.89 for *HmTpi* and *P* = 0.29 for *HmKettin*). However, in contrast to the effect of *Wolbachia*, the expression of *HmTpi*, but not *HmKettin*, was slightly downregulated in males rather than in females. Although this finding may be an artifact of our experiments, we still cannot exclude the possibility that OGVs somehow overactivated dosage compensation in males resulting in the reduced expression of Z-linked *Tpi* genes; hence, further assessment is warranted.

### Only early MK *Wolbachia* affected expression patterns of the *masc* gene.

**FIG 4:**
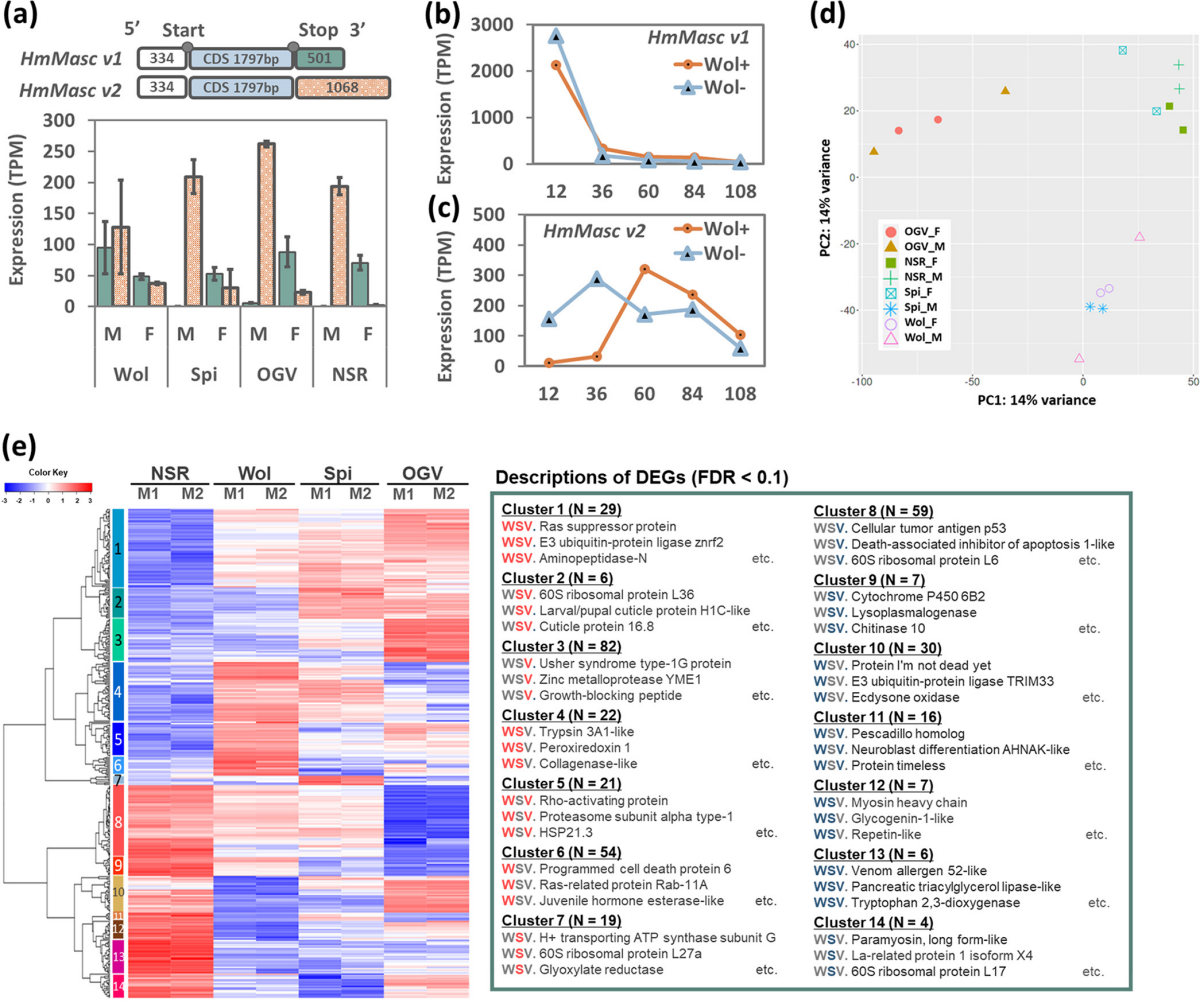
Gene expression patterns in *H. magnanima* harboring male killers. (a to c) Structures and expression levels of *HmMasc* splice variants in embryos. (a) Expression levels of *HmMasc* splice variants in male and female mature embryos (108 hpo). The numbers inside the bars represent base pairs (bp). Both variants had identical 5′ untranslated regions (UTRs; white) and coding sequences (blue); however, their 3′ UTRs (green, *HmMasc v1*; orange, *HmMasc v2*) differed. In the bar plot, the bar with green and orange indicate *HmMasc v1* and *HmMasc v2*, respectively. M, male; F, female; Wol, W^T12^; Spi, Spi^+^; OGV, L. (b and c) Expression dynamics of *HmMasc* variants in the embryos (mixture of sex-undetermined 200 to 300 embryos) of *w*Hm-t-positive (W^T12^) and negative (NSR) lines. The expression levels were quantified every 24 h from 12 to 108 hpo. Panels a to c are shown on different scales due to the different expression levels throughout embryogenesis. (d) Principal-component analysis of gene expression levels in mature embryos (108 hpo). (e) Classification of DEGs in each *H. magnanima* line harboring male killers. DEG expression patterns are classified into 14 clusters. The red and blue colors indicate upregulation and downregulation, respectively, between W^T12^, S^+^, and L males and NSR males.

The dosage compensation system is tightly associated with the sex-determination cascade in several insects (14, 30–42). The *masculinizer* gene (*masc*) is considered an important factor regulating the dosage compensation as well as *dsx* splicing in lepidopteran insects (41). In *B. mori* males (ZZ), *masc* activates the male-type BmDSX protein that promotes male development, and the degraded *masc* expression leads the female-type BmDSX protein in females (ZW) (41). MK *Wolbachia* in Ostrinia furnacalis disrupts dosage compensation and *dsx* splicing by degrading Masc in males (14, 42). We therefore tested whether the MK microbes affect the expression levels of the *masc* gene in *H. magnanima* with different manners by RNA-seq using sex-determined mature embryos (108 hpo, 2 replicates under each condition). Although expression levels were low in 108-hpo embryos, two *masc* isoforms (*HmMasc v1* and *v2*) were expressed in *H. magnanima* (Fig. 4a), which is similar to the results reported by Herran et al. (48), where Ostrinia scapulalis showed sex-specific alternative splicing of the *masc* gene altered by MK *Wolbachia* infection. In the *Spiroplasma* sHm-infected, OGV-infected, and NSR lines, *HmMasc v1* was expressed in females but showed little expression in males, while *HmMasc v2* was more abundant in males than in females (Fig. 4a). Conversely, *w*Hm-t-infected male embryos tended to express higher levels of *HmMasc v1* and lower levels of *HmMasc v2* (Fig. 4a). To further clarify the effects of *w*Hm-t on the expression of *masc*, we compared time-dependent RNA-seq data of both *w*Hm-t-infected and uninfected *H. magnanima* pooled embryos (consisted of approximately 100 to 150 males and females) at 12, 36, 60, 84, and 108 hpo. The expression levels of *HmMasc v1* were highest at the early embryogenesis stage (12 hpo) (Fig. 4b) and decreased as embryogenesis proceeded. At late embryogenesis stages (108 hpo), expression levels of *HmMasc v1* were similar to the expression levels shown in Fig. 4a. Notably, *w*Hm-t infection reduced the abundance of *HmMasc v1* at 12 hpo *H. magnanima* embryos and altered the expression dynamics of the *HmMasc v2* through embryogenesis stages (Fig. 4b and c).

In *H. magnanima*, early MK *Wolbachia* and *Spiroplasma* impaired sex-determination cascades by altering *dsx* splicing, but only *Wolbachia* affected the dosage compensation and expression levels of two *masc* variants. Therefore, our findings suggest that MK *Wolbachia* and *Spiroplasma* alter *dsx* splicing in *H. magnanima* through different machinery. We speculate that MK *Wolbachia* generally alters *dsx* splicing by targeting the *masc* gene in lepidopteran insects, whereas MK *Spiroplasma* alters *dsx* splicing by targeting another factor, such as downstream components of the pathway.

### MK microbes alter the expression patterns of genes involved in metabolism, endocrinology, detoxification, and stress response in different manners.

Through pairwise comparisons among eight groups of pooled 108-hpo embryos (uninfected, *w*Hm-t-infected, *Spiroplasma*-infected, and OGV-infected males or females; two replicates), we found that the three male killers altered gene expression patterns in different manners at the late embryogenesis stage compared with those of the uninfected NSR males (Fig. 4; see Table S1 and S2 in the supplemental material). For example, *w*Hm-t specifically upregulated more Z-linked genes (accounting for 33 out of 140 differentially expressed genes [DEGs]), such as autophagy-protein 5 and multidrug resistance-associated protein, than *Spiroplasma* (8 out of 71) and OGV (5 out of 102) (Table S2) in males, which was probably due to the impaired dosage compensation. In addition, *Spiroplasma* specifically upregulated more numbers of ribosome-associated genes (*n* = 8) in males, whereas OGVs downregulated 10 ribosome-associated genes, suggesting impacts on the protein synthesis (Table S1 and S2; Fig. 4e). Principal-component analysis (PCA) revealed that the expression patterns of *H. magnanima* differed largely depending on microbe infections (Fig. 4d). Intriguingly, the expression pattern of *Spiroplasma*-infected females was similar to that of NSR lines, while the expression pattern of *Spiroplasma*-infected males was similar to that of *Wolbachia*-infected lines. The males harboring early MK *Wolbachia w*Hm-t and *Spiroplasma s*Hm shared more numbers of stress response-associated DEGs (false-discovery rate [FDR], <0.1), such as peroxiredoxin 1, glutathione *S*-transferase 2, and various heat shock proteins (Fig. 4e; Table S2). As mentioned prior, we identified that *S. ixodetis s*Hm and *Wolbachia w*Hm-t both induced female-type *dsx* splicing but affected the dosage compensation system differently in *H. magnanima*. The observed gene expression patterns of *H. magnanima* may reflect similarities and differences in the effects of *Wolbachia* and *Spiroplasma* on the host. In addition, males harboring each microbe shared DEGs involved in stress responses (e.g., glutathione *S*-transferase 1), endocrine systems (e.g., Kruppel homolog 2), morphogenesis (e.g., cuticle proteins and chitinase), metabolism (e.g., aminopeptidase-N) (Table S1), and signal responses (e.g., Ras suppressor protein) (Fig. 4e; Table S2). These results indicate common and specific host responses to each MK microbe.

### MK *Wolbachia* and *Spiroplasma*, but not OGVs, caused abnormal DNA damage during embryogenesis.

**FIG 5:**
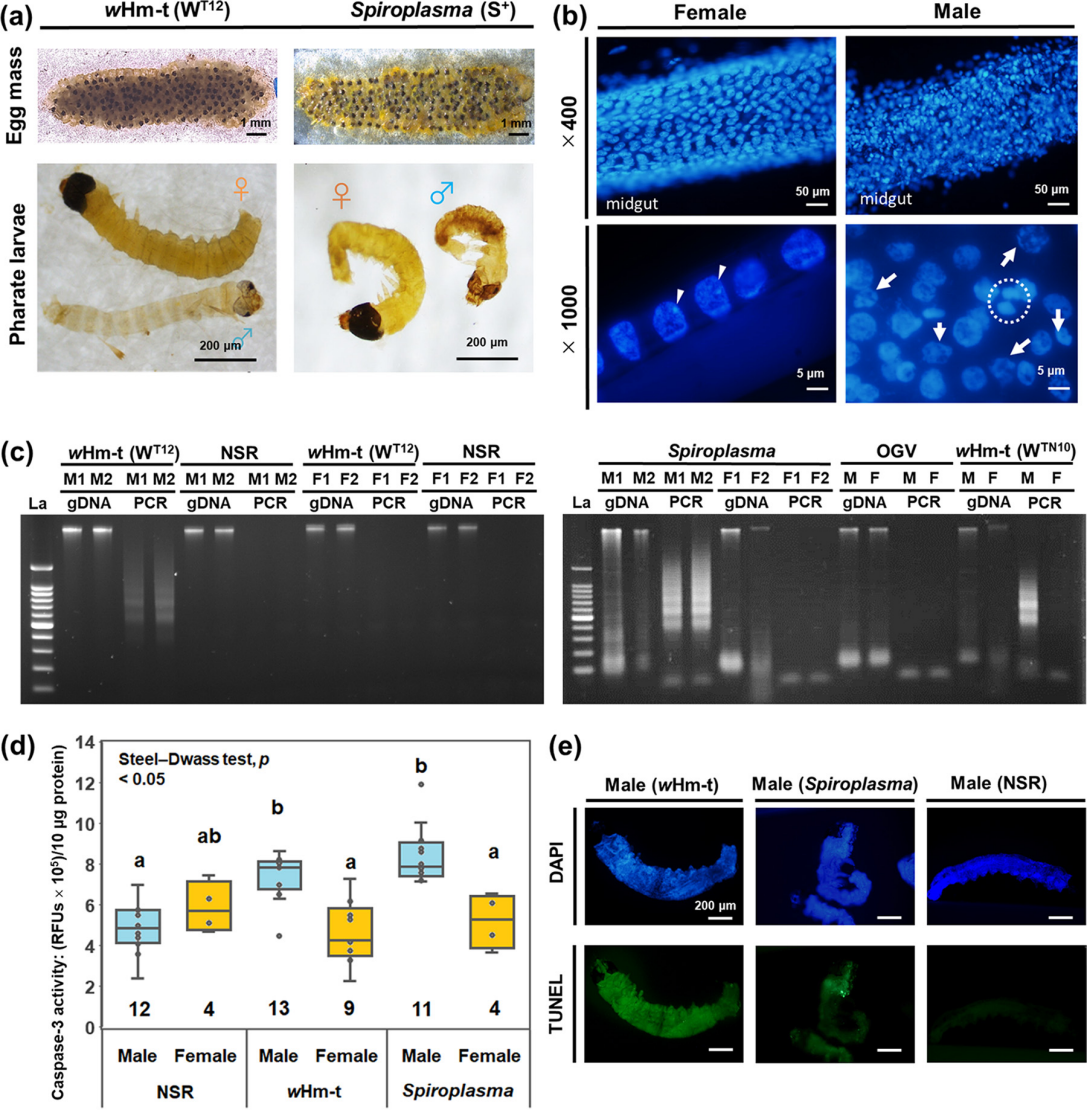
Male-killing *Wolbachia* and *Spiroplasma* caused abnormal apoptosis specifically in *H. magnanima* males. (a) Morphological characteristics of embryos (132 hpo) extracted from W^T12^ and S^+^ egg masses. Male embryos were thinner and more fragile than female embryos. (b) Representative image of the midgut of a W^T12^ embryo (132 hpo) stained with DAPI. Male embryos had abnormally shaped condensed nuclei (white arrows). The white arrowheads indicate heterochromatin (W chromosome). The broken white circle highlights the presumed chromatin bridge (magnification of ×1,000). (c) DNA ladders in the host lines. Genomic DNA (gDNA) and amplicons of laddered DNA (shown as “PCR”) from W^T12^, S^+^, L, and NSR embryos (132 hpo). La, 100-bp DNA ladder; M, male; F, female. (d) Caspase-3 activities in different host lines for NSR, W^T12^ (*w*Hm-t-positive), and S^+^ (*Spiroplasma*-positive) embryos (132 hpo). The numbers inside the bars indicate replicates. Different letters indicate significant differences between groups (Steel-Dwass test, *P < *0.05). (e) TUNEL assays with whole-mounted NSR, W^T12^, and S^+^ male embryos (108 hpo). Green fluorescence indicates apoptosis, and blue fluorescence indicates nuclei counterstained with DAPI. The sex of the embryos was confirmed by detecting the presence or absence of heterochromatin (W chromosome).

Male embryos infected with *w*Hm-t or *Spiroplasma* (132 hpo) were fragile and exhibited nuclear condensation (Fig. 5a and b). We therefore aimed to determine whether male killers underwent abnormal apoptosis during embryogenesis. Nuclear degradation was observed specifically in males (but not females) infected with either *w*Hm-t or *Spiroplasma* (132 hpo) (Fig. 5c). The NSR and L lines harboring OGVs did not show DNA fragmentation. The activities of caspase-3 (an apoptosis effector) were higher (Steel-Dwass test, *P < *0.05) in *w*Hm-t-infected males (73,539.9 ± 3,625.8, relative fluorescence units [RFUs], mean ± SD) and *Spiroplasma*-infected males (84,434.8 ± 3,773.8) than those in noninfected males (48,995 ± 3,773.8), *w*Hm-t-infected females (46,697.9 ± 4,357.6), and *Spiroplasma*-infected females (52,128 ± 6,536.4) (132 hpo) (Fig. 5d). Moreover, terminal deoxynucleotidyltransferase-mediated dUTP-biotin nick end labeling (TUNEL) assays confirmed that males infected with *w*Hm-t or *Spiroplasma* exhibited abnormal nuclear segmentation (108 and 132 hpo) (Fig. 5e; see Fig. S2 in the supplemental material).

### How do microbes kill *H. magnanima* male insects?

**FIG 6:**
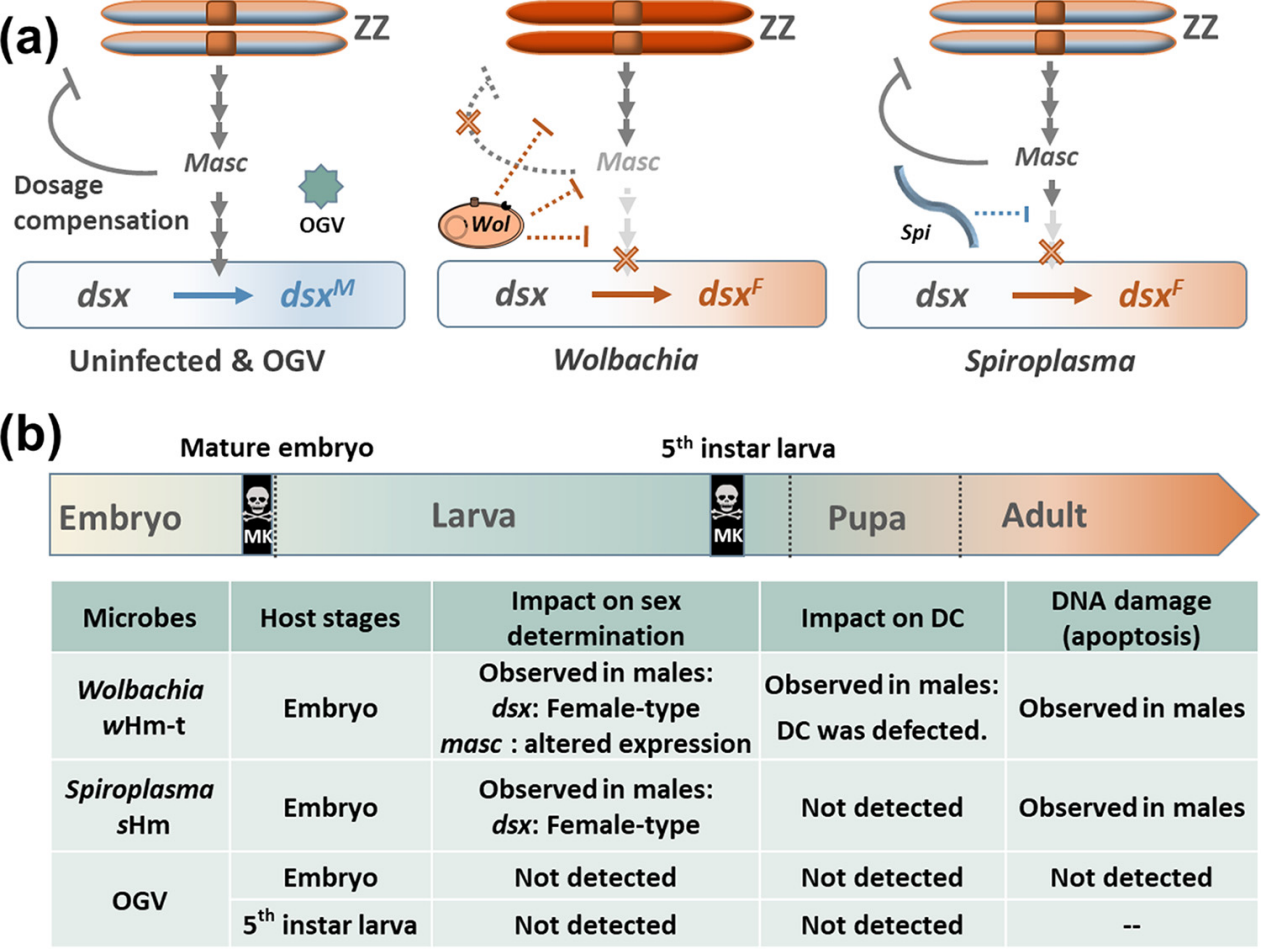
Summary of the effects of male killers on *H. magnanima.* (a) Proposed model for explaining the effects of male killers on the host sex-related machinery. The MK *Wolbachia* affected sex-determination cascades and the dosage-compensation machinery, whereas *Spiroplasma* only affected sex determination in *H. magnanima*. *Wolbachia* possibly affects the dosage-compensation system and sex-determination cascade separately via different mechanisms or by targeting only the *Masc* gene and its upstream cascades, as predicted by Fukui et al. (14). In contrast, *Spiroplasma* utilizes a distinct but unknown mechanism that affects the sex-determination cascade. (b) Summary of the effects of male killers.

It has been hypothesized that microbes accomplish MK by targeting any molecular mechanisms involved in sex determination and differentiation (5, 13–20). One question is whether the mechanisms are shared or specific to each microbe. In this study, we revealed the effects of three male killers on sex determination, the dosage compensation system, and the development of *H. magnanima* (Fig. 6). *Wolbachia* strain *w*Hm-t specifically impaired the male dosage compensation system. Moreover, *Wolbachia* strain *w*Hm-t and *Spiroplasma* strain *s*Hm altered *dsx* splicing and triggered abnormal apoptosis in males. In contrast, late-MK OGVs did not impair host sex-determination cascades during embryogenesis and larval stages.

Improper dosage compensation is considered a direct cause of *Wolbachia*-induced embryonic MK in *Ostrinia* moths (14). Our finding suggests that MK *Wolbachia* generally affects the dosage compensation in lepidopteran insects. The failure of dosage compensation triggers a differential expression of the Z chromosomal genes, which probably affects the viability of *Wolbachia*-infected *H. magnanima* males. However, considering that *Spiroplasma* did not alter the expression of Z-linked genes (and *masc* gene), a failure of dosage compensation may not be the only cause of MK. In *Drosophila*, MK *S. poulsonii* does not impair the dosage compensation itself but induces X chromosome-specific DNA damage by targeting the dosage compensation complex (DCC), which controls the dosage compensation machinery (17–20). Although it is unclear whether *H. magnanima* has some factor(s) corresponding to *Drosophila* DCC, *S. ixodetis s*Hm may induce MK by damaging the dosage-compensated Z chromosome without affecting the dosage compensation itself. In contrast to the early MK *Wolbachia* and *Spiroplasma*, OGVs killed larval or pupal males (12, 35, 36) and did not affect the sex-determination cascades. Charlat et al. (49) suggested that the sex-determination cascade is not the sole target of MK in insects. The present work supports that prediction and indicates that OGVs have a distinct MK mechanism. OGVs may kill males by utilizing the differences in susceptibility between males and females toward viral factors (e.g., genes or viral loads), damaging male-specific organs (e.g., testes), or impairing factors involved in sex dimorphism (downstream sex determination or maintenance systems, such as hormone synthesis). Our study highlights that microbes achieve MK by a variety of mechanisms even in the same insect species. Considering that MK *Wolbachia w*Hm-t, *S. ixodetis s*Hm, and OGVs do not share any genes (11, 23, 34), our results strongly suggest that microbes have acquired MK abilities independently through different evolutionary processes.

Previous studies reported similarities and differences in the effects induced by MK microbes in different *Drosophila* species. For instance, *S. poulsonii* inhibits neurogenesis and induces male-specific abnormal apoptosis in D. melanogaster (17, 18, 50). *Wolbachia* strain *w*Bif also induces abnormal apoptosis but does not affect neurogenesis in the male hosts of *D. bifasciata* (19). Our study also showed that *Wolbachia* and *Spiroplasma* induced female-type *dsx* and abnormal apoptosis in *H. magnanima* males, while only *Wolbachia* specifically affected the dosage compensation system. Kageyama and Traut (13) predicted that mismatches between the genetic sex (ZZ, male) and phenotypic sex (i.e., *dsx* and subsequent gene expression levels) would affect the viability of males. The *dsx* gene controls subsequent gene expression and sex-dependent characteristics in insects (37–39). In *Homona*, defects in sex-determination cascades caused either by *Wolbachia* or *Spiroplasma* likely lead to mismatches between the genetic sex and phenotypic sex. Our transcriptome analyses suggested that *Wolbachia* and *Spiroplasma* affected the motor functions, endocrine systems, and antioxidative/antiaging activities of males, which may elicit severe adverse effects on early male development. We speculate that *Wolbachia*- or *Spiroplasma*-induced stresses result in a similar outcome of abnormal DNA damage and the death of *H. magnanima* male embryos.

## MATERIALS AND METHODS

### Insects.

In this study, we used four Taiwanese *H. magnanima* lines (W^T12^, W^T24^, W^TN10^, and NSR) and three Japanese *H. magnanima* lines (S^+^, L, and W^c^). The W^T12^, W^T24^, and W^TN10^ lines are all-female matrilines harboring the MK *Wolbachia* (*w*Hm-t) strain. The NSR line is a normal sex ratio line (female:male = 1:1) and is free of intracellular bacteria and OGVs (32). The S^+^ and L lines are all-female lines and harbor MK *S. ixodetis* (*s*Hm) (12, 33, 34) and OGVs (11, 12), respectively. The W^c^ line is a normal sex ratio line (female:male = 1:1) and harbors a non-MK *w*Hm-c strain (40), which is closely related to the MK strain *w*Hm-t but lacks an MK-associated prophage region (23). The Taiwanese *H. magnanima* insects were collected from the Tea Research and Extension Station (Taoyuan City, Taiwan) with permission from the Ministry of Agriculture, Forestry, and Fisheries (permission number 27–Yokohama Shokubou 891 and permission number 29–Yokohama Shokubou 1326) in 2015 and 2017. The insects were reared as described previously (51). The nuclear genetic backgrounds of the host lines were homogenized by mating them with the males of the NSR line for at least 10 generations prior to the subsequent experiments.

### Nucleic acid extraction and sex chromatin observations.

To extract RNA and DNA from the sex-determined mature embryos (108 hpo) (Fig. 1), the genetic sexes of mature embryos were determined by observing W chromosomes as described previously (13, 51). Briefly, mature embryos (108 hpo) were dissected on glass slides with forceps. Malpighian tubules were fixed with methanol-acetic acid (50%, vol/vol) and stained with lactic acetic orcein for W chromosome observations. The remaining tissues not used for sexing were stored in the Isogen II reagent (Nippon Gene; for RNA extraction) or a cell lysis solution (10 mM Tris-HCl, 100 mM EDTA, and 1% SDS (pH 8.0) for DNA extraction) at −80°C until subsequent extraction. In total, 12 male or female mature embryos (108 hpo) were pooled and homogenized in the cell lysis solution or Isogen II reagent. The DNA extraction procedure with cell lysis solution was performed as described by Arai et al. (40, 51). To extract RNA, samples homogenized in 600 μL Isogen II reagent were mixed with 240 μL UltraPure distilled water (Invitrogen) and centrifuged at 12,000 × *g* and 4°C for 15 min. Six hundred microliters of each supernatant was mixed with the same volume of isopropanol to precipitate the RNA; then, the resulting solutions were transferred to EconoSpin columns (Epoch Life Science) and centrifuged at 17,900 × *g* and 4°C for 2 min. The RNAs captured in the column were washed twice with 80% ethanol and eluted in 20 μL UltraPure distilled water (Invitrogen).

Total RNA and DNA were also extracted from adults, egg masses (12 to 108 hpo), and OGV-infected 5th instar larvae, as described by Arai et al. (32). The extracted DNA and RNA were quantified using a Qubit v4.0 fluorometer (Invitrogen) and NanoPhotometer NP80 instrument (Implen) and stored at −80°C until subsequent analysis.

### RACE and detection of the *H. magnanima dsx* gene.

To determine sex-specific splicing variants of the *dsx* gene, 3′ RACE experiments were performed according to the method described by Sugimoto and Ishikawa (15), with several modifications. The RNA samples extracted from sex-determined adults and mature embryos (108 hpo) were reverse transcribed via avian myeloblastosis virus (AMV) reverse transcriptase XL (TaKaRa) using the oligo(dT) adapter primer (Table 1). The resulting cDNA samples were amplified with Hmdsx_long2F targeting the conserved *dsx* sequences (coding sequence [CDS]) (Fig. 2a; Table 1) and adapter primers using KOD-Plus-Ver.2 (Toyobo Co., Ltd.) under the following PCR conditions: 2 min at 94°C, followed by 35 cycles of 10 s at 98°C, 30 s at 68°C, and 30 s at 72°C. Because the 1st PCR did not yield clear band patterns, 1 μL of product of the 1st PCR was subjected to nested PCR with Hmdsx_long4F (designed on the 3′ side from Hmdsx_long2F) (Table 1) and adapter primers using Emerald Amp Max master mix under the following conditions: 2 min at 94°C, followed by 20 cycles of 10 s at 98°C, 30 s at 68°C, and 30 s at 72°C. The band observed in the nested PCR products was purified using the Qiaquick PCR/Gel purification kit (Qiagen). Purified DNA was ligated into the pGEM-T easy vector (Promega, WI) and used to transform Escherichia coli JM109 competent cells. Plasmids extracted from E. coli colonies formed on the Luria broth (LB) agarose plates were sequenced using the 3100 genetic analyzer (Applied Biosystems) according to the method described by Arai et al. (40). Primer sets specific for T7 and SP6 promoters of the pGEM-T easy vector (Table 1) were used for sequencing reactions.

**TABLE 1:** Sequences and related information of the primers used in this study

Target	Gene	Primer name	Primer sequence (5′–3′)	Annealing temp (°C)	Reference
*H. magnanima*	*b-actin*	297f	AACTGGGATGACATGGAGAAGATCTGGC	55	Tsugeno et al. (33)
		1139r	GAGATCCACATCTGCTGGAAGGTGGACAG		
	*Hmdsx*	Hm*dsx* long2F (157–185 nt)	GGCGAAATTCTCCGTCCTCCCGTAGAAAC	66	This study
		Hm*dsx* long3F (200–226 nt)	TGCCTAAAGTGAAAACGCCGAGGAGCC		
		Hm*dsx* long4F (370–399 nt)	GCTGTGGCGTTAGATACCTTGGTTGAGAAC		
		Hm*dsx* Mrev (male, 529–549 nt; female, 611–631 and 628–648 nt)	TGGAGGTCTCTTTTCATCCGG		
	*HmTpi* (DNA)	HmTpi_qPCR_F	GTGGCTCACAGTCTGGAGTC	60	
		HmTpi_qPCR_R	CAGTCTTTCCCGCCTCTCTC		
	*HmTpi* (RNA)	HmTpi_F180702_212	GCTGCGAGTGGGTGATTTTG	60	
		HmTpi_R180702_326	GCGATCACTTTCAGACCCGA		
	*HmKettin*	HmKettin_F5464	CGAACCCTTGCTGTTTGTGG	60	
		Hmkettin_R5652	GAATGCAACGTCGAGCCATC		
	*HmEf-1a*	Hmef1a_F_val1_85	TTTCCAGGGTGGTTGAGCA	60	
		Hmef1a_R_val1_193	CCGTTAAGGAGCTGCGTCG		
Apoptosis detection	24-bp Linker		AGCACTCTCGAGCCTCTCACCGCA	55	Staley et al. (57)
	12-bp Linker		TGCGGTGAGAGG		
Colony PCR	T7		GGTCCAATAAGTGATGAAGAAAC	55	Arai et al. (40)
	SP6		TGGAGTAGCGTTTAATT		
RT-PCR	dT15-3sitesAdaptor		CTGATCTAGAGGTACCGGATCCTTTTTTTTTTTTTTT	50	This study
	Adapter		GGATCCGGTACCTCTAGATCAG		

Sex-specific *dsx* splicing variants were detected according to the procedure described by Sugimoto and Ishikawa (15). Briefly, total RNA (100 to 300 ng) extracted from sex-determined adults or mature embryos (108 hpo) was reverse transcribed using PrimeScript II reverse transcriptase (TaKaRa Bio Inc.) at 30°C for 10 min, 45°C for 60 min, and 70°C for 15 min. cDNA was then amplified using KOD-FX Neo (Toyobo Co., Ltd.) with the Hmdsx_long3F (targeting CDS) (Fig. 2) and Hmdsx_Mrev (targeting exon E) (Fig. 2) primer set (Table 1). The following PCR conditions were used: 94°C for 2 min, followed by 45 cycles of 98°C for 10 s and 68°C for 30 s. The amplicons were electrophoresed on 2.0% agarose Tris-borate-EDTA (TBE) buffer (89 mM Tris-borate, 89 mM boric acid, and 2 mM EDTA) gels.

### RNA sequencing, *de novo* assembly, and transcript quantification.

We used 1.0 μg of the total RNA extracted from W^T12^, S^+^, L, and NSR mature embryos (108 hpo) or W^T12^ and NSR egg masses (12, 36, 60, 84, and 108 hpo) to prepare mRNA-seq libraries. Two biological replicates were prepared for each treatment. First, mRNA was extracted from total RNA using the NEBNext poly(A) mRNA magnetic isolation module (New England BioLabs). The mRNA libraries were constructed with the NEBNext ultra II RNA library prep kit for Illumina (New England BioLabs) following the manufacturer’s protocol to prepare 300-bp RNA fragments for 150-bp paired-end (PE150) analysis. The generated sequence data from each library were trimmed by eliminating (i) adaptor sequences, (ii) reads harboring nondetermined sequences exceeding 10%, and (iii) sequences harboring low-quality nucleotides (Qscore, <5) spanning >50% of the read length by Novogen (Beijing, China). Furthermore, all reads showing the average quality below 30 were removed with Trimmomatic (52). The trimmed reads were assembled *de novo* to generate a transcriptome database for *H. magnanima* using Trinity (53) and NAAC Galaxy with the default parameters. All contigs, showing high homologies to genes of *Wolbachia*, *Spiroplasma*, and OGV based on BLASTn analysis, were removed manually. For quantifying transcript abundances from RNA-seq data, the trimmed reads were aligned to the *de novo*-assembled transcriptome database using Kallisto (54) that generated the normalized read count data (transcripts per million [TPM]) for all *H. magnanima* contigs with approximately 60 to 70% map ratios.

### Analyzing dosage compensation and quantifying genes on the Z chromosome.

The effects of male killers on dosage compensation were verified by measuring gene expression differences, as described by Fukui et al. (14) and Gu et al. (29). To assess fold changes in gene expression levels between males and females, the binary logarithms of TPM differences between males and females of each *H. magnanima* line were calculated. *H. magnanima* contigs were then annotated using the *B. mori* gene sets (55) obtained from KAIKObase (https://kaikobase.dna.affrc.go.jp). The binary logarithms of TPM differences between males and females on *B. mori* chromosomes 1 to 28 were plotted.

Z chromosomal gene expression in *H. magnanima* was quantified using reverse transcription-qPCR. Homologs of the *B. mori* Z chromosomal genes *kettin* and *tpi* were extracted from *H. magnanima de novo*-assembled data by performing a BLASTx search. The primer sequences used to quantify these genes are shown in Table 1. Gene doses were quantified using the DNA extracted from male and female embryos. Next, 100 ng of RNA extracted from 12 male or female mature embryos (108 hpo) was reverse transcribed using PrimeScript II reverse transcriptase (TaKaRa) at 50°C for 30 min, followed by denaturation at 95°C for 5 min. The cDNA was used to quantify relative gene expression levels, with normalization to the control gene elongation factor 1a (*ef1a*) (Table 1). The mean cycle threshold (*C_T_*) values of dual samples were calculated for at least eight replicates, and both Δ*C_T_* (*C_T_*Ave Z gene – *C_T_*Ave ef1a) and ΔΔ*C_T_* (Δ*C_T_*male – Δ*C_T_*fem) values were calculated. The dosage (number of Z chromosomes) was estimated based on the 2^–ΔΔ^*^CT^* method, as described by Sugimoto et al. (16). Z chromosomal gene expression levels were analyzed using the Steel-Dwass test in JMP software v9 (SAS, Cary, NC).

### Quantification of the expression levels of *HmIMP*, *HmPSI*, and *HmMasc*.

Homologs of *imp*, *psi*, and *masc* genes were extracted from the *H. magnanima de novo*-assembled transcriptome database using BLASTx (bit-score, >200) with the *B. mori* protein data sets. The time-dependent gene expression levels of the *HmIMP*, *HmPSI*, and *HmMasc* genes in males and females during embryogenesis (12 to 108 hpo) were plotted using the TPM values calculated using Kallisto (54) as mentioned above.

### DEG analysis.

To identify DEGs, TPM values of each transcript calculated as mentioned above were analyzed on iDep9 (http://bioinformatics.sdstate.edu/idep/). The DEGs of each comparison between MK microbe-infected and the uninfected (NSR) *H. magnanima* males were called using DEseq2 (false discovery rate [FDR] cutoff, <0.1; minimum fold change, >2) and were annotated using blast2go (56) and *B. mori* protein data sets (55). In addition, we confirmed that the DEGs were not derived from the microbe-associated transcripts by BLAST searches against *Wolbachia*, *Spiroplasma*, and OGV genome data. The functions of each gene were annotated based on the *B. mori* gene ontology (GO) data on iDep9. GO enrichment analysis was conducted using all annotated genes as the background.

### Observing apoptosis in mature embryos.

DNA segmentation was visualized, as described by Staley et al. (57), using DNA extracted from the sex-determined W^T12^, S^+^, L, and NSR nearly hatched mature embryos (132 hpo). Briefly, 100 ng of DNA, 1 μL of 24-bp linker (1 nM), 1 μL of 12-bp linker (1 nM) (Table 1), and 5 μL of 2× buffer for T4 DNA ligase (Promega) were mixed and incubated at 55°C for 10 min, cooled down gently to 10°C for 55 min, and then incubated at 10°C for 10 min. The reaction mixture was incubated with 1 μL of T4 DNA ligase (3 U/μL) for 15 min at 25°C. Then, 1.5 μL of ligated reactant (15 ng of DNA) was amplified using *Ex Taq* (TaKaRa) with the following conditions: 72°C for 5 min, 25 cycles of 94°C for 1 min, and 72°C for 3 min. The genomic DNA and PCR amplicons were electrophoresed on 1.5% TBE agarose gels to visualize the ladders.

To quantify caspase-3 activity, the sex-determined W^T12^, S^+^, and NSR nearly hatched mature embryos (132 hpo) were homogenized in 120 μL of 1× lysis buffer (10 mM Tris-HCl (pH 7.5), 100 mM NaCl, 1 mM EDTA, and 0.01% Triton X-100). After centrifugation at 3,000 ×*g* for 5 min, 100 μL of each supernatant or 1× lysis buffer was used as a sample or background control, respectively, for the following assays. Caspase-3 activity was quantified using EnzChek Caspase-3 assay kit number 1 (Invitrogen) following the manufacturer’s protocol. The fluorescence intensity (excitation/emission at ~342 and 441 nm, respectively) was quantified using the 1420 ARVO MX-fla multilabel counter (Perkin Elmer). Caspase activities were analyzed using the Steel-Dwass test in JMP software v9 (SAS, Cary, NC).

To visualize DNA damage by performing TUNEL assays, mature embryos (108 hpo) and midguts of the dissected nearly hatched mature embryo (132 hpo) were fixed with 4% (wt/vol) paraformaldehyde phosphate-buffered saline with Tween 20 (PBST; 137 mM NaCl, 8.1 mM Na_2_HPO_4_, 2.68 KCl, 1.47 KH_2_PO_4_, and 0.05% Tween 20 [pH 7.4] as described in Arai et al. [58]) solution for 15 min, washed twice with PBST for 5 min, incubated with 100 μL proteinase K solution (20 μg/mL) for 20 min at 25°C, and washed twice with PBST for 5 min. Positive controls were treated with 50 μL DNase solution (TaKaRa) for 20 min. The TUNEL assays were performed using the DeadEnd fluorometric TUNEL system (Promega) following the manufacturer’s protocol. The TUNEL-stained samples were immersed in 4′,6-diamidino-2-phenylindole (DAPI) solution (1 μg/mL; Dojindo) for 5 min, washed twice with PBST for counterstaining, and mounted with ProLong diamond antifade mountant. Fluorescence was observed by a BX51 microscope (Olympus, Tokyo, Japan) and DP72 camera (Olympus) with filters U-MNIBA2 (excitation wavelength, 470 to 490 nm; absorption wavelength, 510 to 550 nm) and U-MNU2 (excitation wavelength, 360 to 370 nm; absorption wavelength, 420 nm) using the cellSens imaging software (Olympus). Sexes were determined based on the presence of the W chromosome.

### Benefit sharing.

*H. magnanima* was collected from tea plantations at the Tea Research and Extension Station (Taoyuan City, Taiwan) and imported with permission from the Ministry of Agriculture, Forestry and Fisheries (no. 27–Yokohama Shokubou 891 and no. 297–Yokohama Shokubou 1326). Taiwanese *H. magnanima* was maintained only at Tokyo University of Agriculture and Technology. A research collaboration was developed with scientists from the countries providing genetic samples, all collaborators are included as coauthors, and the results of the research have been shared with the provider communities and the broader scientific community. More broadly, our group is committed to international scientific partnerships, as well as institutional capacity building.

### Data availability.

The *Masc*, *Imp*, *PSI*, and *dsx* sequences of *H. magnanima* were deposited in GenBank under accession numbers LC701633 to LC701646. High-throughput sequencing data are available under accession numbers DRA013555 and PRJDB13118 (BioProject). Assembled data are accessible under PRJDB13118.
