# Supplementary material for: Diverse Molecular Mechanisms Underlying Microbe-Inducing Male Killing in the Moth Homona magnanima
Source: Appl Environ Microbiol. 2023 Apr 26;89(5):e02095-22. doi: 10.1128/aem.02095-22 (PMC10231181; doi:10.1128/aem.02095-22)
Supplement: Supplemental file 2 — Supplemental material. Download aem.02095-22-s0002.pdf, PDF file, 0.8 MB [file aem.02095-22-s0002.pdf]

Supplementary Figures

Diverse molecular mechanisms underlying microbe-inducing male killing in the moth *Homona magnanima*

Arai *et al.*

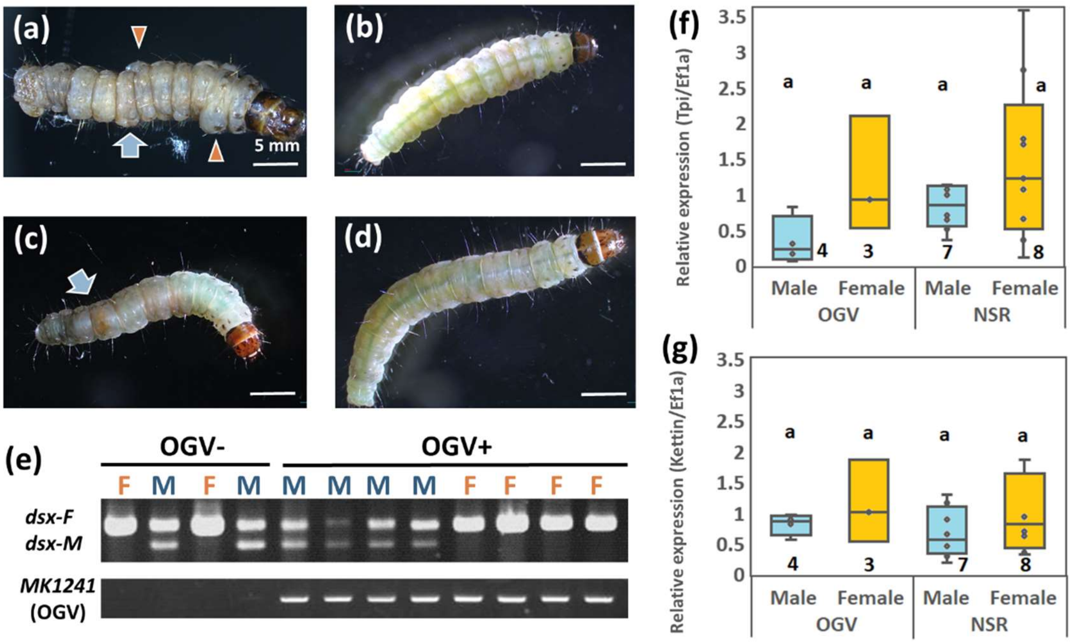

Fig. S1 The OGVs did not affect the host's sex-related machinery during larval development.

(a–d) Morphologies of last-instar larvae of *H. magnanima*. The males harboring OGVs (a) showed carcinoma-like tissue, which was not observed in females of the same line (b), NSR males (c), or NSR females (d). The blue arrows indicate testes, and the orange arrowheads indicate carcinoma-like tissues. (e) The splicing patterns of the *dsx* gene in NSR (OGV–) and L (OGV+) lines. M: males; F: females. (f-g) Expression levels of the Z-linked genes *Tpi* (f) and *Kettin* (g), normalized to expression of the autosomal *Ef1α* gene in the last-instar larvae. The numbers indicate replicates.

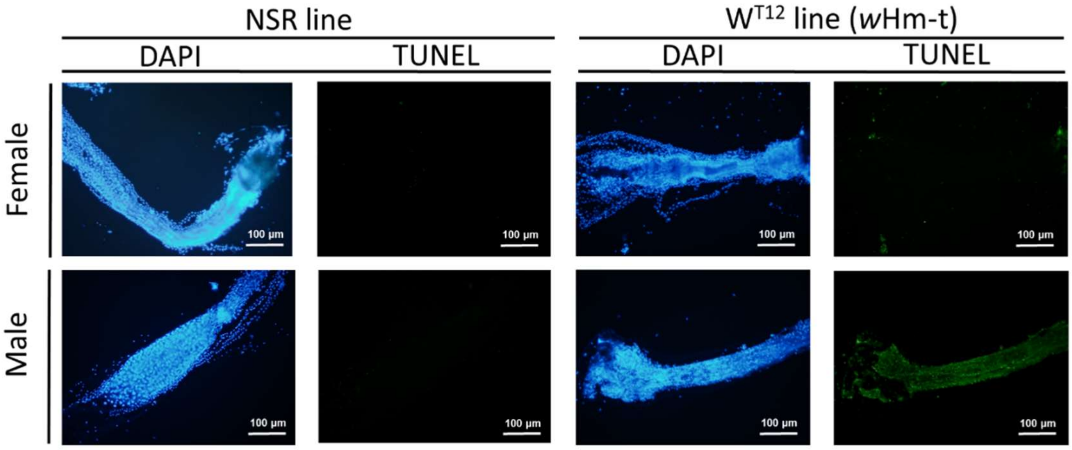

Fig. S2 Apoptosis signals in matured male embryos

TUNEL assays were performed in NSR and W^{T12} tissues (132 hpo males and females) to visualize apoptosis (green). DAPI was used as the counterstain (blue). The sex of each whole-mounted embryo was confirmed after TUNEL staining based on W chromosome observations.
